# 

**Published:** 1900-09

**Authors:** 


### BOOKS RECEIVED.

Practical Urinalysis and Urinary Diagnosis. A Manual for the Use of Physicians, Surgeons and Students. By Charles W. Purdy, LL.D , M. D., Queens University, Fellow of the Royal College of Physicians and Surgeons, Kingston, Canada; Professor of Clinical Medicine at the Chicago Post-Graduate Medical School; Author of "Bright's Disease and Allied Affections of the Kidneys"; also of "Diabetes : Its Causes, Symptoms and Treatment." Fifth revised and enlarged edition. With numerous illustrations, including photo-engravings, colored plates, and tables for estimating total solids from specific gravity, chlorides, phosphates, sulphates, albumin, reaction of proteids, sugar, etc., etc., in urine. 6 x 9 inches. Pages xvi.—406. Philadelphia : F. A. Davis Company, Publishers, 1914–16 Cherry Street. [Price, extra cloth, $3.00 net.]

Manual of Pathology, Including Bacteriology, the Technic of Postmortems, and Methods of Pathologic Research. By W. M. Late Coplin, M. D., Professor of Pathology and Bacteriology, Jefferson Medical College, Philadelphia; Pathologist to Jefferson Medical College Hospital and to the Philadelphia (Blockley) Hospital; Bacteriologist to the Pennsylvania State Board of Health. Third edition, revised and enlarged. 330 illustrations and 7 colored plates. Octavo, 846 pages. Philadelphia: P. Blakiston's Son & Co. [Price, $3.50 net ]

International Clinics. A Quarterly of Clinical Lectures and Especially Prepared Articles on Medicine, Neurology, Surgery, Therapeutics, Obstetrics, Pediatrics, Pathology, Dermatology, Diseases of the Eye, Ear, Nose and Throat, etc. Edited by Henry W. Cattell, A. M., M. D., Director of the Ayer Clinical Laboratory of the Pennsylvania Hospital. With the collaboration of John Ashhurst, Jr., M. D., LL. D., and Charles H. Reed, M. D., of Philadelphia; James T. Whittaker, M. D , LL. D., of Cincinnati. Vol. II. Tenth Series, 1900. Philadelphia: J. B. Lippincott Company. 1900.

Clinical Examination of the Urine and Urinary Diagnosis. A Clinical Guide for the Use of Practitioners and Students of Medicine and Surgery. By J. Bergen Ogden, M D., Instructor in Chemistry, Harvard University Medical School; Assistant in Clinical Pathology, Boston City Hospital, etc. Octavo, pp. 416. Illustrated. Philadelphia: W. B. Saunders & Co. 1900. [Price, $3.00 net.]

Atlas and Epitome of Diseases Caused by Accidents. By Dr. Ed. Golebiewski, of Berlin. Authorised translation from the German, with editorial notes and additions by Pearce Bailey, M. D., Consulting Neurologist to St. Luke's Hospital and the Orthopedic Hospital, New York, and to St. John's Hospital, Yonkers; Assistant in Neurology, Columbia University, etc. Forty colored plates and 143 illustrations in black. Philadelphia: W. B. Saunders & Co. 1900. [Price, $4.00 net.]

Atlas and Epitome of Gynecology. By Dr. Osckar Schaeffer, Privatdocent of Obstetrics and Gynecology in the University of Heidelberg. Authorised translation from the second revised and enlarged German edition. Edited by Richard C. Norris, A. M., M. D., Surgeon-in-charge Preston Retreat, Philadelphia; Gynecologist to the Methodist Episcopal Hospital and to the Philadelphia Hospital, etc. With 207 colored illustrations on 90 plates, and 62 illustrations in the text. Philadelphia: W. B. Saunders & Co. 1900. [Price, $3.00 net.]

A Manual of Personal Hygiene. Edited by Walter L. Pyle, A. M., M. D., Assistant Surgeon to Wills Eye and Ear Hospital, Philadelphia; Fellow of the American Academy of Medicine, etc. 337 pages; illustrated. Philadelphia: W. B. Saunders & Co 1900. [Price, $1.50 net.]

Lessons on the Anatomy, Physiology and Hygiene of Infancy and Childhood. Consisting of Extracts from Lectures given at Rush Medical College, by Alfred C. Cotton, A. M., M. D., Professor of Diseases of Children; Accoucheur and Physician for Diseases of Children, Presbyterian Hospital. 100 illustrations. Chicago: Chicago Medical Book Co. [Price, cloth, $1.50 net.]

Transactions of the Southern Surgical and Gynecological Association, Volume XII. Twelfth session, held at New Orleans, La., December 5, 6 and 7. 1899. W. E. B. Davis, M. D., Secretary. Philadelphia: Wm. J. Dornan, Printer. 1900.

